# Importance-Satisfaction Analysis for Primary Care Physicians’ Perspective on EHRs in Taiwan ^†^

**DOI:** 10.3390/ijerph110606037

**Published:** 2014-06-06

**Authors:** Cheng-Hsun Ho, Hsyien-Chia Wen, Chi-Ming Chu, Yi-Syuan Wu, Jen-Leng Wang

**Affiliations:** 1 Graduate Institute of Information Management, National Taipei University, New Taipei City 23741, Taiwan; E-Mail: jeffher@mail.ntpu.edu.tw; 2 School of Health Care Administration, Taipei Medical University, Taipei 11035, Taiwan; E-Mail: kcukcu@gmail.com; 3 School of Public Health, National Defense Medical Center, Taipei 114, Taiwan; E-Mails: chuchiming@ndmctsgh.edu.tw (C.-M.C.); pu1254@gmail.com (Y.-S.W.)

**Keywords:** electronic health records, primary care physician, importance-satisfaction analysis

## Abstract

The Taiwan government has been promoting Electronic Health Records (EHRs) to primary care physicians. How to extend EHRs adoption rate by measuring physicians’ perspective of importance and performance of EHRs has become one of the critical issues for healthcare organizations. We conducted a comprehensive survey in 2010 in which a total of 1034 questionnaires which were distributed to primary care physicians. The project was sponsored by the Department of Health to accelerate the adoption of EHRs. 556 valid responses were analyzed resulting in a valid response rate of 53.77%. The data were analyzed based on a data-centered analytical framework (5-point Likert scale). The mean of importance and satisfaction of four dimensions were 4.16, 3.44 (installation and maintenance), 4.12, 3.51 (product effectiveness), 4.10, 3.31 (system function) and 4.34, 3.70 (customer service) respectively. This study provided a direction to government by focusing on attributes which physicians found important but were dissatisfied with, to close the gap between actual and expected performance of the EHRs. The authorities should emphasize the potential advantages in meaningful use and provide training programs, conferences, technical assistance and incentives to enhance the national level implementation of EHRs for primary physicians.

## 1. Introduction

**Figure 1:**
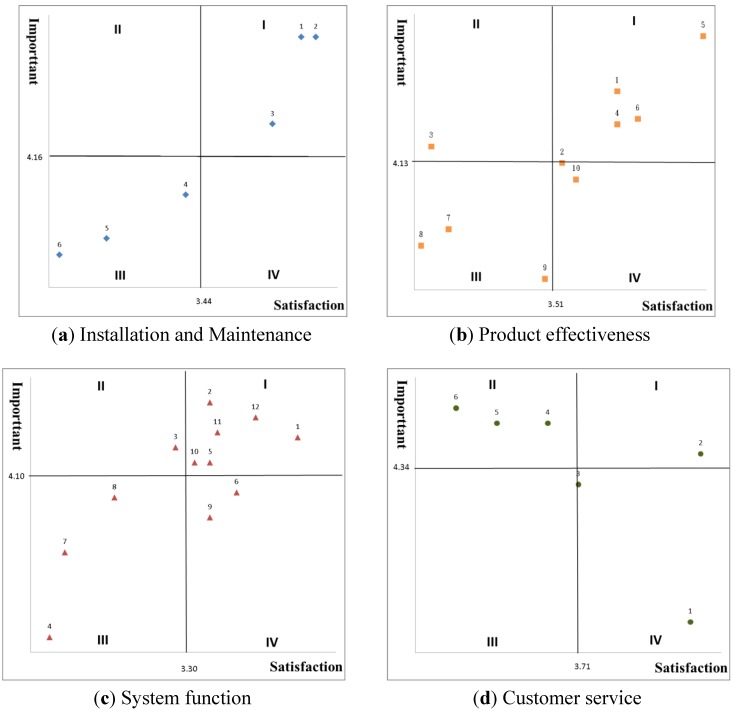
The results of important-satisfaction analysis.

Note: 1. IM: Installation and Maintenance; PE: Product Effectiveness; SF: System Function; CS: Customer Service. 2. I: High Important and High Satisfaction (Keep up the Good Work); II: High Important and Low Satisfaction (Concentrate Here); III: Low Important and Low Satisfaction (Low Priority); IV: Low Important and High Satisfaction (Possible Overkill).

The benefits of the extensive application of information and communication technologies in the medical industry have driven all medical institutions to develop toward using information systems to support their service operations. However, the functioning of health information systems relies on adoption of electronic health records (EHRs). Therefore, promoting EHRs adoption among medical institutions is not only a global trend but also an imperative healthcare policy in all countries. Taiwan’s Ministry of Health and Welfare began a program called “*Accelerating EHRs Adoption in Medical Institutions*” in 2009. The goal of this program was to increase the adoption rate of EHRs among nearly 14,000 clinics nationwide to 70% by 2012. In 2010, the Ministry introduced another program called “Accelerating EHRs Adoption in Clinics” to promote use of an EHRs system that is capable of processing, querying, and exporting data and supporting electronic signature (as shown in Figure 1). About 2,000 basic-level clinics, including general medical clinics, Chinese medicine clinics, and dental clinics, benefited from this program. Because clinics generally have limited labor, financial, and technological resources, they are usually exceptionally careful in evaluating the feasibility of EHRs adoption. Despite the fact that EHRs adoption has been promoted for quite some time, previous research was primarily based on Technology Acceptance Model (TAM) and Theory of Planned Behavior (TPB) and had little coverage of factors affecting EHRs adoption from the importance-satisfaction perspective. 

The purpose of this study is to empirically evaluate the importance of EHRs for clinic physicians and their satisfaction with EHRs and to provide suggestions about promotion of EHRs adoption. To better understand consumers’ post-purchase view of a product or service and identify the strengths and weaknesses of the product or service, Martilla and James developed an analytical technique called Importance-Performance Analysis (IPA) in 1977 [1]. IPA analyzes the attributes of a product or service using a two-dimensional grid that is divided into four quadrants, respectively meaning “Keep up the good work”, “Concentrate here”, “Low priority”, and “Possible overkill”. In this grid, the y-axis shows the level to which users or consumers view the attribute(s) of the product or service as important, whereas the x-axis indicates the performance of the product or service on the attribute(s) perceived by its users or consumers. The explanation for each quadrant is as follows: The first quadrant named “Keep up the good work” suggests high importance and high performance. The attributes in this quadrant are important, and the management’s job is to continue to perform well in these areas. The second quadrant named “Concentrate here” indicates high importance but low performance, suggesting improvement efforts should be concentrated here. The third quadrant named “Low priority” indicates low importance and low performance. Improving performance on attributes in this area is not a primary or necessary task. The fourth quadrant named “Possible overkill” contains attributes of low importance but high performance. Managers may have been overly concerned about their performance on these attributes and should adjust their effort on them. Ideally, attributes should be distributed along the straight line extending from bottom left to top right of the chart. Attributes in the second quadrant should be given a higher priority; attributes in the first quadrant should be given the same priority as before; attributes in the fourth quadrant should be given a lower priority, and resources allocated for these attributes should be moved to attributes that fall in the second quadrant. Yang evaluated service performance by customer satisfaction with an importance-satisfaction model [2]. As the objective of this paper is to explore factors affecting EHRs adoption from user perspective, the factors will also be evaluated based on user satisfaction. In O’Sullivan [3], the IPA chart is divided into four quadrants based on medians on the two axes. Chaven, Ewert, and Magill proposed that attributes can be better differentiated if the chart is divided by the means of actual performance and importance levels [4]. It can be found from the above literature that IPA has been applied in a wide array of service areas. By evaluating the strengths and weaknesses, this technique can help management improve and develop effective strategies. Therefore, this study also employs this technique with the performance dimension replaced by satisfaction in an attempt to capture the relation between importance and satisfaction with regard to EHRs adoption. The perspectives of clinic physicians on Installation and Maintenance, Product Effectiveness, System Function and Customer Service of EHRs were collected. After the empirically comparison, we can find what attributes of EHRs clinic physicians saw as important but were not satisfied with, and the study could further provide a direction for improving the acceptance of EHRs.

## 2. Methods

### 2.1. Sample Frame

The Taiwanese government has been promoting Electronic Health Records adoption by promoted primary care physicians since 2000. Therefore, how to extend EHRs adoption rate by measuring physicians’ perspective of importance and performance of EHRs has become one of the critical issues for healthcare organizations. This study conducted a comprehensive survey consisted of 1034 general medical clinics that participated in the 2010 Program of Accelerating EHRs Adoption in Clinics launched by Department of Health. 

### 2.2. Importance-Performance Analysis

The applications of IPA to evaluation of healthcare services are numerous. Yavas and Shemwell used this technique to analyze hospital competitiveness from customer perspective [5]. Dolinsky and Caputo applied it to explore the importance of healthcare attributes for members of health maintenance organizations (HMO) and performance of competitors on these attributes [6]. Among domestic studies, Weng *et al.* stated in a study of relationship quality and relational benefit that IPA is an effective tool for analyzing service quality and healthcare marketing [7]. Liu used this technique to evaluate the importance of attributes of the health insurance policy for citizens and their satisfaction with the policy [8]. The author also provided suggestions as to how to improve attributes of high importance but low satisfaction. Tsai, Kung, Weng, and Shih analyzed the perceptual gap in service quality between physicians and citizens [9]. They used IPA to find the differences in service quality perception between providers and recipients of medical services and further obtain a direction for improving the service quality of clinics. Lo, Liu, and Lin also performed an IPA to examine the gap between the actual performance of chain health checkup clinics and the performance expected by their members [10]. Their goal was to assist management in developing service strategies. Yeh focused on perceptual gap in service quality between patients with coronary artery disease and nursing staff. Through an IPA, the author explored factors affecting patient satisfaction and obtained suggestions as to how to improve the service quality of cardiac catheterization [11].

IPA answers two basic questions: (1) How important is a certain product/service attribute to customers? (2) How satisfied are customers with the firm’s performance on this attribute? The key advantage of IPA is the “synergistic effect of their simultaneous examination” of customers’ views of the importance of key attributes of the product/service being studied and their satisfaction with the performance of these attributes. The value of IPA approach lies in identifying relative, rather than absolute, levels of the vertical and horizontal axes on the grid. Since Martilla and James’ [1] work, IPA has increasingly become a useful research technique for organizations to identify the strengths and weaknesses of their market offerings and to develop effective strategies. This study is based on a data-centered analytical framework that Martilla and James [1] suggested. In order to avoid distorting interpretation of findings, we adapted our labels: “high important and high satisfaction”, “high important and low satisfaction”, “low important and high satisfaction” and “low important and low satisfaction”. However, Martilla and James’ IPA is a low-cost and easily understood technique that can yield important insights, and its purpose is to help users increase their usefulness in making strategic decisions [1]. Therefore, the original perception labels were put in parenthesis of four quadrants.

### 2.3. Measurements and Procedures

The research instrument was a self-administered structured questionnaire developed according to Yang [2]. Skulmoski, Hartman, and Krahn [12] observe that a homogeneous group needs a smaller sample and recommend purposive sampling for expert recruitment. In this study, eight representative experts, including three professors of medical affairs management and information management and five physicians, are identified by three criteria required for “expertise”: (1) knowledge and experience with the issues of investigation; (2) capacity and willingness to participate; (3) sufficient time to participate in the Delphi discussion.

The first step is typically qualitative in nature in order that a wide range of views may be elicited. Open-ended questions are important since the resulting data are the basis for closed-end questionnaires in subsequent rounds. In view of the time limitations of discussion technique, the study combined some specific criteria from literature reviews and open-ended questions. The questionnaire items were modified according to expert opinions and evaluated for importance, adequacy, and clarity. All of the items had a content validity index greater than 0.8. 

Later, a pilot study was conducted on physicians from 30 clinics to ensure that each item was semantically appropriate. Measurement reliability was assessed using Cronbach’s alpha. The internal consistencies of all variables are considered acceptable since they exceed 0.80, thus signifying satisfactory reliability. The final questionnaire consisted of 34 items in four dimensions, including six items in “Installation and maintenance”, 10 in “Product effectiveness”, 12 in “System function”, and six in “Customer service”. All of the items were measured in a structured format on a 5-point Likert-type scale, ranging from 1 (very important/satisfaction) to 5 (very unimportant/dissatisfaction). The distribution of responses was presented by standard deviation in Table 3. The mean importance and satisfaction ratings of 68 EHRs attributes by four dimensions respectively were plotted as 34 points on the importance-performance grid in Figure 1. It showed mean values presented different from each other given the distribution. A copy of the questionnaire in this study is attached in appendix.

This questionnaire was administered to 1034 clinics via mail. A total of 581 surveys were ultimately completed. Of the total number of surveys, twenty five were considered problematic and excluded because of excessive missing data which more than one of four dimensions of important/satisfaction was blank. Finally, 556 valid responses were analyzed, resulting in a valid response rate of 53.77%.

## 3. Results

This study drew on a sample of 556 responses to evaluate the importance of EHRs for clinic physicians and their satisfaction with EHRs. To test the difference between respondents and the population, the chi-square goodness-of-fit test was performed on three variables, including “department”, “number of physicians”, and “health insurance region”. The *p*-values for these three variables were 0.14, 0.122, and 0.144 respectively, all of which were above the level of significance, indicating no significant difference between the sample and the population in these three variables. 

The descriptive statistics of the sample (Table 1) were as follows: Most responses were contributed by clinics classified in the Taipei region in the health insurance system (150 responses or 27%). Responses contributed by clinics in the central region (120 responses or 21.6%) and the Kaohsiung and Pingtung region (113 responses or 20.3%) respectively formed the second and third largest groups. In terms of department, responses contributed by clinics of internal medicine constituted the majority (216 responses or 38.8%) followed by those collected from surgery clinics (180 responses or 32.4%). Unlike US and other western countries, Taiwan’s primary care allows patients to visit specialists directly such as Surgical, OBS & GYN and Pediatric without referral from general practitioners. Most respondents were in the age groups of 40~49 (264 persons or 47.5%) and 50~64 (226 persons or 40.6%). Five hundred and ten (510) of the respondents were male (91.7%), and only 46 were female (8.3%). In terms of position, most respondents served as the director of their clinics (519 persons or 93.3%). 32 respondents were partnered physicians (5.8%), and only five respondents were employed physicians (0.9%). Most clinics were run as a sole proprietorship (333 clinics or 59.9%), and 219 clinics reported having 2~5 physicians (39.4%). In the aspect of history, most clinics had been in business for 11~20 years (206 clinics or 37.1%). Clinics in business for 6~10 years (187 clinics or 33.6%) and those in business for no more than five years (104 clinics or 18.7%) respectively formed the second and third largest groups. The statistics of monthly volume of outpatient visits showed 211 clinics had 1000~1999 visits (37.9%) followed by 157 clinics with 2000~2999 visits (28.2%) and 121 clinics with no more than 999 visits (21.8%). 

**Table 1:** Demographic data of respondents

Variable	Number	%		Variable	Number	%
**Region**				**Proprietorship**		
Taipei	150	27		Solo	333	59.9
Northern	60	10.8		2–5	219	39.4
Central	120	21.6		5–9	2	0.4
Southern	99	17.8		10 & above	2	0.4
Kaohsiung & Pingtung	113	20.3		**Business (years)**		
Eastern	14	2.5		5	104	18.7
**Specialty**				6–10	187	33.6
Medical	216	38.8		11–20	206	37.1
Surgical	180	32.4		21 & above	59	10.6
OBS & GYN	13	2.3				
Pediatric	88	15.8		**Volume (month)**		
Multiple	59	10.6		Below 999	121	21.8
**Age**				1000–1999	211	37.9
Below 39	54	9.7		2000–2999	157	28.2
40–49	264	47.5		3000 & above	67	12.1
50–64	226	40.6				
65 & above	12	2.2				
**Sex**						
Male	510	91.7				
Female	46	8.3				
**Position (Title)**						
Director	519	93.3				
Partner	32	5.8				
Employed	5	0.9				

In order to understand the relationship between importance and satisfaction, this study used Pearson’s product-moment correlation technique to examine the relevance among attributes (Table 2). Results showed that satisfaction with customer service (CS) was significantly and positively correlated with satisfaction with installation and maintenance (IM) (r = 0.626), product effectiveness (PE) (r = 0.651) and system function (SF) (r = 0.679). Importance of CS and importance of SF were positively related to the level of significance (r = 0.653). Satisfaction with SF was significantly and positively related to satisfaction with IM (r = 0.730) and PE (r = 0.746). Importance of SF was significantly and positively related to importance of IM (r = 0.675) and that of PE (r = 0.715). The relation between satisfaction with PE and satisfaction with IM was significantly positive (r = 0.719), so was that between importance of PE and importance of IM (r = 0.684). However, the correlations between importance and satisfaction of four dimensions, ranging 0.324 (IM), 0.311 (PE), 0.256 (PE), 0.235 (CS), were not highly correlated. The result might demonstrate there was a gap between perceived satisfaction and perceived importance of each dimension.

**Table 2:** The Pearson correlations between variables

Variables	Imp. of IM	Sat. of IM	Imp. of PE	Sat. of PE	Imp. of SF	Sat. of SF	Imp. of CS
Satisfaction of IM	0.324 **						
Importance of PE	0.684 **	0.293 **					
Satisfaction of PE	0.199 **	0.719 **	0.311 **				
Importance of SF	0.675 **	0.284 **	0.715 **	0.283 **			
Satisfaction of SF	0.186 **	0.730 **	0.214 **	0.746 **	0.256 **		
Importance of CS	0.596 **	0.199 **	0.584 **	0.096 *	0.653 **	0.080	
Satisfaction of CS	0.199 **	0.626 **	0.228 **	0.651 **	0.283 **	0.679 **	0.235 **

Note: Imp. = Importance; Sat. = Satisfaction; IM = Installation and Maintenance; PE = Product Effectiveness; SF = System Function; CS = Customer Service; * *p* < 0.05; ** *p* < 0.01; *** *p* < 0.001.

Therefore, we used IPA approach that Martilla and James suggested to capture the relationship between importance and satisfaction ratings among attributes of EHRs adoption. This study plotted the results on a grid of four quadrants divided by the overall means of importance and satisfaction. The distribution of the attributes on the grid is shown in Table 3. Among the attributes in the dimension of “Installation and maintenance”, three attributes fall into the first quadrant (High Important and High Satisfaction) and no attribute is placed in the second quadrant (High Important and Low Satisfaction). The three attributes for which effort should be maintained are: Avoid affecting medical operations during service hours (IM1), Avoid affecting stability of the existing information systems (IM2), and provide training on use of the system functions (IM3). In the dimension of “Product effectiveness”, four attributes are located in the first quadrant, including: Simplify the administrative procedure in health records management (PE1), Reduce the cost of human resources for health history management (PE4), Save the health history storage space (PE5), and Meet the global trend of energy saving and carbon reduction (PE6). However, one attribute falls into the second quadrant. It is Simplify National Health Insurance (NHI)’s random review of claims for insurance payment (PE3), such as by not using paper. Among the attributes in the “System function” dimension, The function of the electronic signature (attribute: SF1), The efficiency of creating electronic signature (SF2), The function of querying electronic health records (SF5), The availability of multiple data backup methods (such as remote backup and import to portable drives) (SF10), The responsiveness of system operations (SF11), and The availability of an easy-to-understand user interface (SF12) are attributes that fall in the first quadrant. The function of checking abnormal conditions of electronic signatures (SF3) is an attribute located in the second quadrant. Finally, in the dimension of “Customer service”, Customer service staff’s familiarity with the EHRs system (CS2) is only one attribute in the first quadrants; three attributes including Customer service staff capable of solving hardware/software troubles of the system (CS4), Customer service staff’s efficiency of solving troubles (CS5), and Customer service staff’s efficiency of restoring normal operation of the system (CS6) fall into the second quadrant. 

**Table 3:** Importance and satisfaction analysis

Dimensions	Attributes	Importance Mean (SD)	Importance Ranking	Satisfaction Mean (SD)	Satisfaction Ranking	Located Area
Installation & Maintenance	IM1	4.36 (0.79)	1	3.70 (0.94)	2	I
	IM2	4.36 (0.78)	1	3.74 (0.94)	1	I
	IM3	4.20 (0.82)	3	3.62 (0.96)	3	I
	IM4	4.07 (0.87)	4	3.38 (1.09)	4	III
	IM5	3.99 (0.96)	5	3.16 (1.04)	5	III
	IM6	3.96 (1.02)	6	3.03 (1.11)	6	III
	Mean	4.16 (0.70)		3.44 (0.88)		
Product Effectiveness	PE1	4.26 (0.77)	2	3.69 (0.94)	3	I
	PE2	4.13 (0.82)	6	3.53 (1.05)	6	I
	PE3	4.16 (0.83)	5	3.15 (1.50)	9	II
	PE4	4.20 (0.82)	4	3.69 (0.98)	3	I
	PE5	4.36 (0.78)	1	3.94 (0.95)	1	I
	PE6	4.21 (0.83)	3	3.75 (1.01)	2	I
	PE7	4.01 (0.86)	8	3.20 (1.42)	8	III
	PE8	3.98 (0.91)	9	3.12 (1.51)	10	III
	PE9	3.92 (0. 94)	10	3.48 (1.0)	7	III
	PE10	4.10 (0.91)	7	3.57 (1.01)	5	IV
	Mean	4.12 (0.68)		3.51 (0.86)		
System Function	SF1	4.18 (0.79)	4	3.60 (0.93)	1	I
	SF2	4.25 (0.81)	1	3.37 (1.08)	5	I
	SF3	4.16 (0.81)	5	3.28 (1.10)	9	II
	SF4	3.78 (0.98)	12	2.95 (1.48)	12	III
	SF5	4.13 (0.80)	6	3.37 (1.05)	5	I
	SF6	4.07 (0.81)	8	3.44 (1.02)	3	IV
	SF7	3.95 (0.86)	11	2.99 (1.45)	11	III
	SF8	4.06 (0.83)	9	3.12 (1.32)	10	III
	SF9	4.02 (0.85)	10	3.37 (1.14)	5	IV
	SF10	4.13 (0.84)	6	3.33 (1.27)	8	II
	SF11	4.19 (0.83)	3	3.39 (1.05)	4	I
	SF12	4.22 (0.78)	2	3.49 (1.0)	2	I
	Mean	4.10 (0.70)		3.31 (0.93)		
Customer Service	CS1	4.24 (0.79)	6	3.82 (0.93)	2	IV
	CS2	4.35 (0.76)	4	3.83 (0.94)	1	I
	CS3	4.33 (0.78)	5	3.71 (1.0)	3	IV
	CS4	4.37 (0.79)	2	3.68 (1.20)	4	II
	CS5	4.37 (0.79)	2	3.63 (1.25)	5	II
	CS6	4.38 (0.78)	1	3.59 (1.26)	6	II
	Mean	4.34 (0.75)		3.70 (0.93)		

Note: IM = Installation and Maintenance; PE = Product Effectiveness; SF =System Function; CS = Customer Service; SD: Standard deviation.

Among the attributes of “Installation and maintenance”, three attributes are located in the third quadrant (Low Importance and Low Satisfaction) and no attribute is located in the fourth quadrant (Low Importance and High Satisfaction) on Figure 1. The three attributes that should be given a lower priority are: Supervise and audit training on administrative procedures (IM4). The cost of system installation is reasonable (IM5), and the cost of system maintenance is reasonable (IM6). Among the attributes of “Product effectiveness”, two attributes should be given a lower priority: Integrate inter-hospital data and provide continuous healthcare (PE7) and Acquire data from other hospitals to avoid repeated examinations, inspections and medication (PE8). Additionally, two attributes may have been overly emphasized (Low Importance and High Satisfaction): Simplify NHI’s supervisory and auditing procedures (PE2) and Improve the confidentiality of health records (PE10). In the dimension of “System function”, three attributes are plotted in the third quadrant: Multiple ways to create electronic signatures (SF4), Patients’ health records can be exported and imported individually or in batch (SF7), Patients’ health records can be extracted to meet the random review by NHI (SF8). Two attributes are located in the fourth quadrant: Preserve and present the history of changes in each patient’s health records (SF6) and Support access control (SF9). Finally, among the attributes of “Customer service”, no attribute falls into the third quadrant and two attributes are located in the fourth quadrant. The two attributes that may have been overly emphasized are Customer service staff’s service attitude (CS1) and Customer service staff's familiarity with regulations about EHRs (CS3).

## 4. Discussion

Results showed that most clinic physicians considered simplification of the administrative procedure in health records management, reduction of cost of human resources for health records management, reduction of data storage space, and compliance with the global trend of energy saving and carbon reduction as critical to EHRs adoption. They also showed high satisfaction with their EHRs system in these aspects. 

The extant research of EHRs indicated that advanced EHRs technologies are positively related to treatment effects. Advanced EHRs technologies can help avoid unnecessary or repeated examinations and radioactive examinations [13,14,15], reduce cost of labor for managing health records [16], reduce use of paper, and create an additional income by using the released storage space for other purposes. Yasunaga *et al.* investigated the barriers to implementation of EHRs in Japan. In their study, 80% of the hospitals mentioned that the cost of EHRs adoption was high; 45% agreed that the EHRs system could facilitate interactions between hospitals; 25% recognized EHRs as helpful for improving the time effectiveness for physicians [17]. Results of this study are generally consistent with findings of previous research. In this study, user satisfaction with EHRs was found to have a positive relation with user perception of the cost effectiveness of the system. The more that clinic physicians agreed that using EHRs is cost-effective, the most satisfied they would be with the EHRs. Identifying attributes inthe “Concentrate here” quadrant, that is, attributes that are important but have not given enough attention, is one of the goals of this study. This study attempts to find attributes critical to promotion of EHRs adoption. The results can be a reference for the government, clinics, and information system providers. Results indicated that clinic physicians perceived the current insurance claims review and filing operations as important but were not satisfied with the current operations. This is because despite the fact that the current EHRs systems they use support data production, query, and export, they are required to send paper copies of health records or printed health records to the health insurance authority for review. If NHI adopts XML as the standard data format in the future, hospitals and clinics can directly send XML files of CDA R2 (including signatures and time stamps) for review, and the review results (including reasons of approval or rejection, codes, and amount of payment) can be automatically sent back to and written in the health information system. All NHI branches will be more able to meet the various needs of clinics and provide a digital claims review procedure that is simpler, more convenient, and faster. As a result, clinics will show higher intention toward EHRs adoption.

In addition, “Integrate inter-hospital data and provide continuous healthcare” and “Acquire data from other hospitals to avoid repeated examinations, inspections, and medication” in the dimension of product effectiveness and “Patients' health records can be exported and imported individually or in batch” in the dimension of system function were the attributes that clinic physicians identified as unimportant, and the current statuses of these attributes were unsatisfactory. However, this result did not conform to NHI’s goal of promoting use of EHRs, that is, improving the healthcare quality by avoiding repeated medication and examinations. It can be inferred that clinic physicians might have insufficient knowledge about how to exchange health records across medical institutions to avoid repeated examinations and medications and improve the effectiveness of medical resources. Of course, it is also likely that clinics paid less attention to cross-institution integration of health records due to the disparity in benefit of health records exchange. Therefore, in order to improve clinic physicians’ knowledge in this aspect and the uniformity of benefits of exchange of health records, the authorities concerned should provide more education training and develop supplementary measures to support the current policy. Moreover, the Electronic Medical Record Exchange Center should integrate health information exchange mechanisms to facilitate exchange of health data, increase the effectiveness of health data exchange, and avoid duplication of resources. By doing so, the prevalence rate of EHRs, continuity of healthcare, and the healthcare quality can all be effectively improved. Finally, the respondents rated “Avoid affecting medical operations during service hours” and “Avoid affecting stability of the existing information systems” as important and were satisfied with their system in these two aspects. In other words, the clinic physicians recognized the importance of and trusted “the supplier of their current medical information systems”. Hence, if the authorities concerned continues to appropriate funds for promotion of EHRs and rely on suppliers of medical information systems for promotion and implementation of EHRs, its EHRs adoption policy might be achieved more easily. 

To increase EHR adoption, United States government will spend approximately $30 billion in incentives for physicians [18]. To be eligible for such incentive payments, physicians must use EHRs in a meaningful manner, exchange electronic health information to improve the quality of care, and report on clinical quality and other measures [19]. On the other hand, there is the threat of reduced payments for those that fails to comply. Thus, the aim behind is the meaningful use in US is to improve quality and efficiency of care by encouraging clinicians and hospitals to use EHRs and exchange information. [20] Likewise in US, the Department of Health (DOH) in Taiwan intensively focusing on meaningful use of EHRs from last few years. However, in Taiwan there are no such incentives offered by the DOH specifically for meaningful use. Fortunately, Taiwan DOH is the only buyer under the National Health Insurance system. Clinic physicians were required to submit their claims via computer, so the government can facilitate the policy and procedures to promote, and enhance the intention of physicians to adopt EHRs. In addition, information technology vendors in Taiwan can fully support their needs and deliver service promptly. The lesson we can learn from US is that the direct incentives embedded in the Health Information Technology for Economic and Clinical Health Act may have a positive influence on EHRs adoption especially for hospitals with high Medicare and/or Medicaid caseloads [21]. Therefore, the government subsidy policy for primacy physician should be continued to extend the adoption of EHRs in clinics. Also, for enhancing clinic physician adoption behavior, like US had set up many “regional extension center” for EHRs promotion, more training and education programs should be promoted. [22].

In this study, the subjects were limited to general clinics targeted by National Health Insurance’s program, so the results might be generalizable only to clinics that have adopted EHRs. Future researches can achieve a higher generalizability of the results by increasing the sample size or including a wider area to be sampled. This study analyzed attributes affecting EHRs adoption using the importance and satisfaction technique. As adoption of innovations and technologies can be examined from other perspectives as well, future research is suggested to compare different theories to find a better explanation of the EHRs adoption behavior. 

## 5. Conclusions

We found what EHRs attributes Taiwanese clinic physicians saw as important but were not satisfied with, such as Insurance claims filing and review operations; the function of checking abnormal conditions of electronic signatures; customer service staff capable of solving hardware/software problems of the system; customer service staff’s efficiency of solving troubles; customer service staff’s efficiency of restoring normal operation of the system. Therefore, this study provided a direction to policy makers by focusing on attributes which physicians found important but were dissatisfied with, to close the gap between actual and expected performance of the EHRs. Also, the authorities should emphasize its potential advantages in meaningful use and provide incentives, technical assistance and training programs, conferences to enhance the national level implementation of EHRs for primary care physicians.

## Appendix A. Measurements of Constructs

To understand the cognition of using the EHRs in your work, please answer the following questions.

According to your using of EHRs, please give a check mark on the blank of Importance from 1 (Very Important) to 5 (Not Important at all) and Satisfaction (Very Satisfied) to 5 (Very dissatisfied) appropriately.

### Part I. Cognition of EHRs (Importance and Satisfaction)

#### Installation and Maintenance (IM)

IM1 Avoid affecting medical operations during service hours
IM2 Avoid affecting stability of the existing information systems
IM3 Provide training on use of the system functions
IM4 Supervise and audit training on administrative procedures
IM5 The cost of system installation is reasonable
IM6 The cost of system maintenance is reasonable

#### Product Effectiveness (PE)

PM1 Simplify the administrative procedure in health records management
PM2 Simplify NHI’s supervisory and auditing procedures
PM3 Simplify NHI’s random review of claims for insurance payment
PM4 Reduce the cost of human resources for health history management
PM5 Save the health history storage space
PM6 Meet the global trend of energy saving and carbon reduction
PM7 Integrate inter-hospital data and provide continuous healthcare
PM8 Acquire data from other hospitals to avoid repeated examinations, inspections and medication
PM9 Improve the professional image of the clinic
PM10 Improve the confidentiality of health records

#### System Function (SF)

SF1 The function of electronic signature
SF2 The efficiency of electronic signature
SF3 The function of checking abnormal conditions of electronic signatures
SF4 Multiple ways to create electronic signatures
SF5 The function of querying electronic health records
SF6 Preserve and present the history of changes in each patient’s health records
SF7 Patients’ health records can be exported and imported individually or in batch
SF8 Patients’ health records can be extracted to meet the random review by NHI
SF9 Support access control
SF10 The availability of multiple data backup methods
SF11 The responsiveness of system operations
SF12 The availability of an easy-to-understand user interface

#### Customer Service (CS)

CS1 Customer service staff’s service attitude
CS2 Customer service staff's familiarity with functions of the EHRs system
CS3 Customer service staff's familiarity with regulations about EHRs
CS4 Customer service staff capable of solving hardware/software troubles of the system
CS5 Customer service staff’s efficiency of solving troubles
CS6 Customer service staff’s efficiency of restoring normal operation of the system

### Part II. Personal Information

1. Region: □Taipei □Northern   □Central   □Southern   □Kaohsiung & Pingtung   □Eastern
2. Sex: □ Male   □ Female
3. Age: □below 39  □40–49    □50–64   □65 & above
4. Specialty: □Medical   □Surgical   □Pediatric   □OBS & GYN    □Multiple:__
5. Position (Title): □director   □partner     □Employed
6. Number of physicians: □solo     □2–5     □5–9     □above 10
7. Years of service: □below 5 years   □6–10 years   □11–20 years   □21 & above
8. Patient visit per month: □below 999   □1000–1999   □2000–2999   □ 3000& above
